# *In Vitro*, *Ex Vivo*, and *In Vivo* Evaluation of Nanoparticle-Based Topical Formulation Against *Candida albicans* Infection

**DOI:** 10.3389/fphar.2022.909851

**Published:** 2022-07-08

**Authors:** Sajid Khan Sadozai, Saeed Ahmad Khan, Abdul Baseer, Rooh Ullah, Alam Zeb, Marc Schneider

**Affiliations:** ^1^ Department of Pharmacy, Kohat University of Science and Technology, Kohat, Pakistan; ^2^ Department of Pharmacy, Abasyn University, Peshawar, Pakistan; ^3^ Riphah Institute of Pharmaceutical Sciences, Riphah International University, Islamabad, Pakistan; ^4^ Department of Pharmacy, Biopharmaceutics and Pharmaceutical Technology, Saarland University, Saarbrücken, Germany

**Keywords:** Ketoconazole, PLGA, topical gel, sustained release, *Candida albicans*

## Abstract

Ketoconazole is commonly used in the treatment of topical fungal infections. The therapy requires frequent application for several weeks. Systemic side effects, allergic reactions, and prolonged treatment are often associated with non-compliance and therapy failure. Hence, we developed an optimized topical antifungal gel that can prolong the release of drug, reduce systemic absorption, enhance its therapeutic effect, and improve patient compliance. Ketoconazole-loaded PLGA nanoparticles were prepared by the emulsion/solvent evaporation method and were characterized with respect to colloidal properties, surface morphology, and drug entrapment efficiency. The optimized ketoconazole-loaded PLGA nanoparticles and commercially available silver nanoparticles were incorporated into a Carbopol 934P-NF gel base. This arrangement was characterized and compared with commercially available 2% ketoconazole cream to assess physical characteristics of the gel, *in vitro* drug release, *ex vivo* skin permeation and retention, and *in vivo* studies on Wister male albino rats. The results showed that polymeric PLGA nanoparticles were very effective in extending the release of ketoconazole in our optimized formulation. Nanoparticles were smooth, spherical in shape, and below 200 nm in size which is consistent with the data obtained from light scattering and SEM images. The *ex vivo* data showed that our gel formulation could strongly reduce drug permeation through the skin, and more than 60% of the drug was retained on the upper surface of the skin in contrast to 38.42% of the commercial cream. The *in vivo* studies showed that gel formulation could effectively treat the infection. This study demonstrates that our topical gel could be effective in sustaining the release of drug and suggests its potential use as a possible strategy to combat antifungal-resistant *Candida albicans*.

## 1 Introduction

Superficial fungal infections are very common throughout the world, and incidences are increasing day by day. The prevalence of fungal skin infections comprises 20–25% of the world population (Buil et al., 2020). Superficial fungal infections are keratinophilic in nature. They increase in the keratinized layers of the skin, hair, and nails (Borgers et al., 2005). They are predominately caused by the dermatophyte members of three common genera: *Microsporum*, *Trichophyton*, and *Epidermophyton*. *C. albicans* and other non-albicans species mostly cause superficial fungal infections. The severity of the infection depends upon the site and the involvement of fungal species (Sharifzadeh et al., 2013; Cleveland et al., 2015; Klingspor et al., 2015). In various studies, it was reported that candidiasis was treated by several compounds of different classes, either belonging to polyenes, azoles, echinocandins, nucleoside analogs, or allylamines. Their efficacy depends on the type, site of infection, and susceptibility of the *Candida* species (Pfaller et al., 2013; Pappas et al., 2016). Geographical and environmental conditions play a vital role in the causative agent and spread of fungal skin infections. Superficial fungal infections are more common in tropical regions where the temperature is warm and humid. Similarly, the chance of spread is more in low socioeconomic conditions and densely populated areas where opportunities for skin-to-skin contact are more, with poor hygienic conditions (Coates et al., 2020).

A topical drug delivery system is administered on the skin’s outer surface to treat local dermatological conditions (Ashara et al., 2014). They are used when local action is desired rather than a systemic effect. The drug remains on the skin or may penetrate the dermis through the epidermal layers, but is not absorbed in the systemic circulation (Choi and Maibach, 2005). The topical dosage form is formulated to achieve maximum therapeutic effect locally by incorporating active agents, protective agents, adsorbents, cleansing agents, emollients, viscosity builders, and stabilizers (Choudhury et al., 2017). A topical drug delivery system has numerous advantages over oral and injectable systems. Therefore, researchers are focusing on developing such drug delivery systems to avoid the problems associated with other routes of administration (Borgheti-Cardoso et al., 2016). The topical dosage form is applied to the affected area only, and it by-passes the first-pass metabolism and releases the drug directly to the site where it is needed (Mayba and Gooderham, 2018). It can be easily administered, with no need for any special skills or help of another person for administration (Eastman et al., 2014). The topical dosage form is applied on the skin to deliver the drug directly to the area to be treated, thus proving prolonged localized action without frequent administration (Waghule et al., 2019).

Various antifungal agents are commercially available in conventional topical dosage forms like creams, lotions, and sprays. The problem with these conventional topical dosage forms is that they require frequent applications for several weeks until infection signs and symptoms completely disappear. Systemic side effects, as well as local side effects, are associated with it. Thus, conventional topical dosage forms are often inappropriate and might be considered inconvenient by the patient, resulting in therapy failure (Firooz et al., 2015).

Topical ketoconazole is one of the most often used azole drugs, commercially available as 2% cream and ointment (Andriole, 2000). Based on its hydrophobic nature, it belongs to Biopharmaceutics Classification System (BCS) class II drug. Ketoconazole has limited bioavailability and poor aqueous solubility (Sodeifian et al., 2021). Therefore, a higher amount of drug is required to achieve the desired antifungal activity. The mechanism of action of the azole group is to inhibit the 14α-sterol enzyme encoded by the ERG11 gene, involved in the biosynthesis of fungal cell membrane sterol ergosterol. Some species of non-candida albicans are naturally resistant to azoles, causing therapy failure when used against these species. It was reported that *Candida* had the ability to develop high-level resistance against azoles (Oxman et al., 2010; Fothergill et al., 2014). Additionally, it can cause side effects such as allergic and severe skin reactions. Similarly, prolonged therapy is required to achieve the desired results. To overcome these problems, a combination of available drugs with the blend of modern technology is required to overcome the aforementioned problems.

In nanomedicines, silver nanoparticles (AgNPs) have gained incredible popularity in recent years. AgNPs are used in different fields for different purposes (Monteiro et al., 2012). Silver’s biological efficacy has been known for centuries, but their assessment as AgNPs on a scientific basis has received tremendous attention in recent years (Rasheed et al., 2017). AgNPs and specifically the released silver ions can inhibit the replication of microbial agents, including bacteria and fungi, by disrupting cellular membranes and other organelles (Tang and Zheng, 2018). AgNPs attributed synergistic effects against various resistant species when combined with antibiotics (Zhang et al., 2014; Liao et al., 2019; Sadozai et al., 2020).

Improving the efficacy of available antimicrobials against resistant strains is a key concern for future treatments. Recently in various studies, AgNPs showed promising results effectively killing antibiotic-resistant microorganisms (Scandorieiro et al., 2016; Patra and Baek, 2017; Wang et al., 2020). AgNPs could be used in combination with existing antimicrobials to enhance their effectiveness and reverse antimicrobial resistance (Pfalzgraff et al., 2018; McNeilly et al., 2021). In a recent study, biogenic silver nanoparticles in combination with fluconazole or metronidazole showed synergistic effects and reduced the effects of anticandidal agents (Aabed and Mohammed, 2021). Similarly, in another study, AgNPs in combination with antibiotics showed strong synergistic effects against *E. coli*, *P. aeruginosa*, and *S. aureus* (Deng et al., 2016). It was also reported that minimum inhibitory concentration (MIC) of β-lactam antibiotics against *E. coli* was reduced several folds when used in combination with AgNPs (Patra and Baek, 2017). It was also reported that AgNPs enhanced the activity of ampicillin, tetracycline, streptomycin, and rifampicin (Markowska et al., 2014).

The current study is being designed to overcome the bioavailability issues of ketoconazole. The emulsion/solvent evaporation method was used for the preparation of ketoconazole-loaded poly(lactide-co-glycolide) (PLGA) nanoparticles (<300 nm) that could potentially assemble in wrinkles (Stracke et al., 2006; Schneider et al., 2009) and hair follicles (Lademann et al., 2008) to provide a prolonged release to the skin tissues. The optimized ketoconazole-loaded PLGA nanoparticles and commercially available silver nanoparticles (AgNPs) were incorporated into a Carbopol 934P-NF base gel for topical application. The objective of this study was to sustain the release of ketoconazole at the site of action and reduce its permeation into systemic circulation. Moreover, the optimized gel formulation was evaluated and compared with commercially available cream to effectively treat skin infection induced by the resistant strain of *Candida albicans* in Wister male albino rats.

## 2 Materials and Methods

### 2.1 Materials

Ketoconazole was a kind gift from Bryon Pharmaceuticals Pvt. Ltd., Peshawar, Pakistan. Poly(lactide-co-glycolide) (PLGA) was purchased from Evonik Industries, Darmstadt, Germany. Silver nanoparticles (≤ 20 nm) were purchased from PHORNANO Holding GmbH, Austria. Polyvinyl alcohol (PVA) soluble in cold water was used (Mowiol 4–88, Kuraray Europe, Hattersheim, Germany); Fisher Scientific Chemicals Ltd., United Kingdom, provided dichloromethane. Methanol was purchased from VWR International GmbH (Darmstadt, Germany). Potassium dihydrogen phosphate and di-potassium hydrogen phosphate were purchased from Merck, Germany. Tween^®^ 20 was purchased from Sigma life science. Carbopol 934P-NF and triethanolamine were purchased from Sigma-Aldrich (Steinheim, Germany). Ultra-pure distilled and deionized water was produced with Millipore ultra-pure water system (Milli-Q^®^ Synthesis).

### 2.2 Preparation of Ketoconazole-Loaded PLGA Nanoparticles

As reported previously, ketoconazole-loaded PLGA nanoparticles were prepared by the emulsion/solvent evaporation method (Sadozai et al., 2020). Various formulations were designed, as shown in Table 1. Different drug/polymer ratios were studied to investigate their impact on the physicochemical properties of nanoparticles.

**TABLE 1:** Various formulations of ketoconazole-loaded PLGA nanoparticles.

Formulation	Ketoconazole (mg)	PLGA (mg)	DCM (ml)	2% PVA (ml)	Sonication	
					AMP (%)	Time (sec)
100% keto, 0% PLGA	100	0	2	10	40	90
75% keto, 25% PLGA	75	25	2	10	40	90
50% keto, 50% PLGA	50	50	2	10	40	90
25% keto, 75% PLGA	25	75	2	10	40	90
15% keto, 85% PLGA	15	85	2	10	40	90
12.5% keto, 87.5% PLGA	12.5	87.5	2	10	40	90
0% keto, 100% PLGA	0	100	2	10	40	90

Keto, ketoconazole; PLGA, poly(lactide-co-glycolide); DCM, dichloromethane; PVA, polyvinyl alcohol; AMP, amplitude.

### 2.3 Characterization of Nanoparticles

#### 2.3.1 Dynamic Light Scattering

The mean particle size and polydispersity index (PDI) of nanoparticles were determined by dynamic light scattering (DLS) with a Zetasizer Nano ZS (Malvern Instruments, Malvern, UK) at 25°C with a backscattering angle of 172° as discussed in our previous study (Ashjari et al., 2020; Sadozai et al., 2020).

#### 2.3.2 Scanning Electron Microscopy

Scanning electron microscopy (SEM) was used to determine the morphology, particle size, and size distribution of nanoparticles (EVO HD 15, Carl Zeiss Microscopy GmbH. Jena, Germany). Samples were coated with a thin layer of gold to provide sufficient conductivity (Q150RES, Quorum Technologies Ltd, East Grinstead, United Kingdom). Micrographs were taken with an acceleration voltage of 5 kV (Sa-Barreto et al., 2017).

#### 2.3.3 % Entrapment Efficiency (%EE)

The ketoconazole entrapped in nanoparticles was determined using high-performance liquid chromatography (HPLC) according to an established method with some modifications (Kanaujia et al., 2011; Sadozai et al., 2020). The entrapment efficiency (%EE) was determined indirectly according to a previously reported method (Shafique et al., 2017). Briefly, the supernatant obtained after centrifugation of nanoparticles was collected. The amount of free ketoconazole present in the supernatant was determined analytically and the encapsulation was calculated using the following equation:

$$\mathrm{EE}\%=\frac{Total\;drug\;added\;\left(mg\right)-Free\;drug\;in\;\sup erna\tan t\left(mg\right)}{\mathrm{Total}\;\mathrm{drug}\;\mathrm{added}\;\left(\mathrm{mg}\right)}X\;\mathrm{100}$$ (1)

The drug loading was calculated using the entrapped drug amount (total drug–free drug) with respect to the formulation weight:

$$\text{Drug}\;\text{loading}=\frac{\mathrm{Drug}\;\mathrm{entrapped}\;\left(\mathrm{mg}\right)}{\mathrm{Weight}\;\mathrm{of}\;\mathrm{dried}\;\mathrm{powder}\;\left(\mathrm{mg}\right)}\text{X}\;100$$ (2)

#### 2.3.4 Drug Release Studies

The drug release from nanoparticles was studied in phosphate buffered saline (PBS) pH 7.4 at 37°C. Tween-20 2.0% w/v was added to the dissolution medium to obtain sink conditions since ketoconazole is insoluble in PBS pH 7.4; briefly, 10 mg of drug-loaded nanoparticles were dispersed in 50 ml dissolution medium and stirred continuously at 37°C. 1.0 ml aliquots were withdrawn at predetermined intervals and centrifuged at 24,000 RCF. The supernatant was collected, and the pellets were redispersed in a 1.0-ml dissolution medium and then returned to the main tube (Kataoka et al., 2019). The amount of ketoconazole in the samples was analyzed using the established HPLC method.

### 2.4 Gel Preparation

#### 2.4.1 Preparation of Carbopol 934P-NF Gel Base

Carbopol 934P-NF was selected as a hydrophilic polymer due to its high purity and pharmaceutical grade. Formulations were prepared based on the concentration of Carbopol 934P-NF, as shown in Table 2. Each formulation was prepared by taking a specific amount of Carbopol 934P-NF and adding deionized water in a beaker to make the final volume up to 100 ml. The beaker was placed on a magnetic stirrer for 3 hours to make a slurry. The polymer solution was kept in a dark place overnight for complete swelling. The pH was adjusted to 6.5 by the addition of triethanolamine (Jana et al., 2014). After adjusting the pH, physical characteristics and spreadability studies were carried out to choose the suitable Carbopol gel base.

**TABLE 2:** Various formulations of Carbopol 934P-NF gel base.

Serial No.	Formulation	Carbopol 934P-NF (mg)	Water (ml)	pH
1	1% Carbopol gel	1	100	6.5
2	1.5% Carbopol gel	1.5	100	6.5
3	2% Carbopol gel	2	100	6.5
4	2.5% Carbopol gel	2.5	100	6.5

#### 2.4.2 Incorporation of NPs in Gel

After the selection of a suitable Carbopol 934P-NF gel base, different formulations were prepared w/w by incorporating the optimized formulation of ketoconazole-loaded PLGA nanoparticles, pure ketoconazole nanoparticles without PLGA, silver nanoparticles, optimized ketoconazole-loaded PLGA nanoparticles and silver nanoparticles in combination and pure drug (ketoconazole), as shown in Table 3. All formulations were mixed properly with the help of mortar and pestle to ensure uniform distribution.

**TABLE 3:** Various formulations of Carbopol 934P-NF containing ketoconazole and silver nanoparticles.

Ingredient	Formulations						
	Blank gel	PLGA NP gel	Keto NP gel	Keto PLGA NP gel	Keto-drug gel	AgNP gel	Keto PLGA NP + AgNP gel
Carbopol gel 934P-NF	2%	2%	2%	2%	2%	2%	2%
Blank PLGA NPs	—	16.5%	---	—	—	—	—
Ketoconazole NPs	—		2%	—	—	—	—
Ketoconazole PLGA NPs	—	—	—	*16.5%	—	—	*16.5%
Ketoconazole pure Drug	—	—	—	—	2%	—	—
Silver NPs	—	—	—	—	—	1%	1%

PLGA NPs (100% PLGA NPs), ketoconazole NPs (100% ketoconazole NPs), ketoconazole PLGA NPs (12.5% ketoconazole + 87.5% PLGA NPs) *(16.5% w/v particles correspond to 2% ketoconazole pure drug based on entrapment efficiency).

### 2.5 Evaluation of the Gels

#### 2.5.1 Appearance

All gel formulations’ appearance was inspected visually for color, clarity, and presence of any particulate. This test is important for esthetic point of view as well as patient compliance (Parashar et al., 2013).

#### 2.5.2 pH

pH plays an important role in the preparation of Carbopol gel 934P-NF as it is highly pH-sensitive. The pH of all the gel formulations was measured using Mettler Toledo Seven Compact S230 (Germany) pH meter. About 1 mg sample from all formulations was taken and stirred with distilled water to form a uniform suspension. The volume came up to 50 ml and pH of the suspension was measured (Parashar et al., 2013).

#### 2.5.3 Spreadability

Spreadability is an important parameter to determine the quality of a topical preparation. The therapeutic efficacy depends on the gel’s ability to spread uniformly and easily. Spreadability studies were performed using a stainless-steel apparatus having a lower plate and upper plate. In between these two plates was a sample compartment in which gel was loaded. After loading the gel in this compartment, a transparent plastic scale was set above the steel plate holding the sample in the center. Two glass plates weighing 2 kg were placed above the transparent scale. As the glass plate is placed above it, the gel spreads on the scale. The diameter was measured after 5 minutes. Each sample was measured three times to get the mean value.

#### 2.5.4 Drug Content Determination

For the uniform distribution of the drug in a gel formulation, drug content was determined. The gel’s drug content was determined by accurately weighing 1.0 mg of gel and dissolving it in 40 ml PBS solution containing 2% Tween-20 in a beaker under continuous stirring to make a suspension. This suspension was filtered (0.45-micron filter paper), and the drug content was analyzed by the described HPLC method.

### 2.6 *In Vitro* Drug Release Studies

*In vitro* drug release studies were carried out by using Franz diffusion cells with a spherical diameter of 25 mm and a diffusion area of 3.56 cm^2^. The diffusion was investigated across a cellulose membrane with a thickness of 4.44 mm and pore size of 12–14,000 Da was placed between the lower cell reservoir and glass cell top containing the sample. The receiving compartment was filled with a solution of PBS and 2% Tween-20 with a pH adjusted to 7.4 and temperature maintained at 32.0 ± 0.5°C with a magnetic stirrer. 0.1 mg of each sample was applied evenly on the Teflon sheet, having a diameter of 14 mm which was placed above the cellulose membrane in the donor compartment. After predetermined intervals (1, 2, 4, 8, 12, and 24 h), 400 µL receptor fluid was withdrawn from the receiving compartment and was replaced with the same volume of fresh solution (Bhaskar et al., 2009). Samples were analyzed on RP-HPLC to determine the *in vitro* drug release from the Carbopol gel.

### 2.7 *Ex Vivo* Studies

#### 2.7.1 Skin Permeation Studies

Excised human skin as the gold standard for *ex vivo* studies was used. The human abdominal skin from Caucasian female patients was obtained from Caritas Klinik, Lebach, Germany. The procedure was approved by the Ethical Committee of the Aerztekammer des Saarlandes, Saarbrücken, Germany (Code 204/08, 22 December 2008). The fatty tissue attached to the epidermis was removed, carefully washed with water, and stored in the refrigerator. From the stored skin, circular samples with a diameter of 25 mm were punched out with the help of a plunger and hammer. The epidermis was thoroughly washed with water and allowed to hydrate for 1 h before being mounted on the Franz diffusion cells with the stratum corneum (S.C.) facing the donor compartment (Luengo et al., 2006; Luengo et al., 2021). Drug delivery from the topical dosage form is an important parameter in developing a formulation, to determine how much drug is released or permeated through the skin to reach the dermis and potentially the systemic circulation.

#### 2.7.2 Tape Stripping Method

The tape stripping method was employed to determine the amount of drug present in the uppermost layer of the skin stratum corneum (Luengo et al., 2021) to treat the superficial infection of the skin. After 24 h, the skin was removed from the Franz diffusion cell. The skin surface was washed 10 times with a cotton swab to remove the extra amount of gel present on the skin’s surface. The skin was then placed on a flat surface and fixed with the help of pins at the corners of the skin so that the stratum corneum faced upward. Modified Scotch Magic Tape was used to remove the stratum corneum from the skin completely. A tape strip was applied on the skin, and after application, a roller was rolled with a uniform force above it in two opposite directions, and the tape strip was removed with the help of forceps. The same procedure was repeated 12 times to ensure complete removal of stratum corneum, which was also checked with the help of Squame scan, which is used to scan the stratum carenum protein content on the tape strips (Hahn et al., 2010). The first two strips were discarded, and the remaining ten were mixed with mono isopropyl amine methanol (2:500) and shaken to dissolve adhered ketoconazole in the solvent mixture. Then, the solvent mixture was allowed to evaporate the organic mixture on a magnetic stirrer. The release medium was added to dissolve the drug and filtered through a 0.45-μm membrane filter. The filtrate was analyzed for drug concentration by RP-HPLC as discussed earlier (Escobar-Chávez et al., 2008; Clausen et al., 2016).

#### 2.7.3 Drug Retention Studies

In addition to determining the amount of drug present on SC, the percentage of drug penetrated was also analyzed by washing the skin 10 times with a cotton swab and cutting the skin into small pieces, and the skin was homogenized in the solvent mixture for 2 h to extract the drug. The resulting solution was centrifuged for 10 min at 2795 RCF, and the supernatant was analyzed on RP-HPLC with the same procedure described earlier (Minghetti et al., 2006).

### 2.8 *In Vivo* Studies

The *in vivo* studies were carried out according to the guidelines approved by the ethical committee of the Department of Pharmacy, KUST vide notification number “Ref NO./KUST/Ethical Committee/2286”.

#### 2.8.1 Experimental Design

Wistar albino male rats (230–250 mg) were obtained from PCSIR laboratories in Peshawar. All the animals were acclimatized under standard animal house conditions for 7 days before starting the *in vivo* studies. The animals were randomly divided into four groups, each group containing six Wistar albino healthy male rats. Group-I was treated with keto-drug gel, 2% Carbopol gel, and 2% pure ketoconazole drug as a positive control. Group-II was considered negative control and treated with a blank gel containing 2% Carbopol gel without any drug. Group-III was treated with test gel keto PLGA NPs and AgNPs containing 2% Carbopol and 2% optimized Keto PLGA nanoparticles combined with AgNPs. Group-IV was treated with commercially available 2% ketoconazole cream considered as the reference group. After inoculation with a fungal infection, all the animals were treated once a day for 7 days, respectively.

#### 2.8.2 Preparation of *Candida albicans* Strain

The *C. albicans* strain was obtained from Shifa International Hospital Pathology Department (ATCC-10231) and was allowed to grow for 48 h at 30°C on sabouraud dextrose agar media. The cells were collected, washed, and suspended in sterile saline to get 10^7^ CFU/ml (Barros et al., 2007).

#### 2.8.3 Induction of Fungal Infection

All rats were prepared by removing the hair on the dorsal area approximately 2 cm^2^ with the help of a razor or electrical hair trimming machine. Each rat was intradermally injected with 100 µl of *C. albicans* strain having a concentration of 10^7^ CFU/ml. The injected area was rubbed with cotton to avoid edema. The fungal infection was observed after 3 days of inoculation in the affected area (Araújo et al., 2009).

#### 2.8.4 Clinical Investigations

All the animals were clinically examined periodically at days 0, 4, and 7 in a week to check the symptoms such as rashes, red or purple patches, white patches over the affected area, scaling, cracking, pimples filled with puss, hair loss, and the treatment efficacy will be scored from 0 to 5 according to a modification of the reported clinical parameters as shown in Table 4 (Aggarwal et al., 2013).

**TABLE 4:** Clinical parameters and therapeutic efficacy.

Serial no.	Clinical parameter	Score
1	No sign of infection	0
2	Slight erythematous skin	1
3	Redness on a well-defined area with swelling, bald patches, and scaly area	2
4	Large areas with redness and ulceration	3
5	Loss of hair and partial damage to the skin	4
6	Excessive damage to the skin with complete hair loss	5

### 2.9 Statistical Analysis

The results were expressed as mean ± standard deviation using different statistical tests. The results were compared by one-way analysis of variance (ANOVA). The data were considered significantly different at *p*-value ˂ 0.05 by using Graph Pad Prism software (Version 5).

## 3 Results and Discussion

To overcome the challenges faced in conventional drug delivery systems, a topical nanocarrier drug delivery system emerged as a suitable alternative (Díaz and Vivas-Mejia, 2013). The advantages of nanocarrier as a drug delivery system are high surface-to-volume ratio, nanoscale size, and easy fabrication with controlled physicochemical properties. Topical nanocarrier as a drug delivery system enhances the aqueous solubility of hydrophobic drugs. They can extend shelf life, prolong and control the release of drug (Lavan et al., 2003), improve the stability against moisture (Lockman et al., 2002), modify the pharmacodynamics and pharmacokinetics of the drug, and reduces the side effects by targeting the specific site at the cellular level (Allen and Cullis, 2004). The objective of this study was to design and fabricate a topical gel preparation that can prolong the release of ketoconazole, reduce its systemic absorption, and improve its therapeutic potential against resistant *C. albicans* strain when combined with AgNPs.

### 3.1 Characterization of Nanoparticles

#### 3.1.1 Physicochemical Properties of Nanoparticles

**FIGURE 1:**
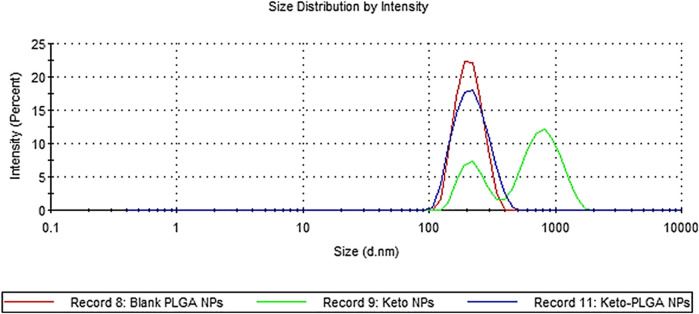
Particle size distribution by intensity. Blank PLGA NPs contain no drug, keto PLGA NPs contain 12.5% ketoconazole and 87.5% PLGA, and keto NPs contain 100% ketoconazole.

**FIGURE 2:**
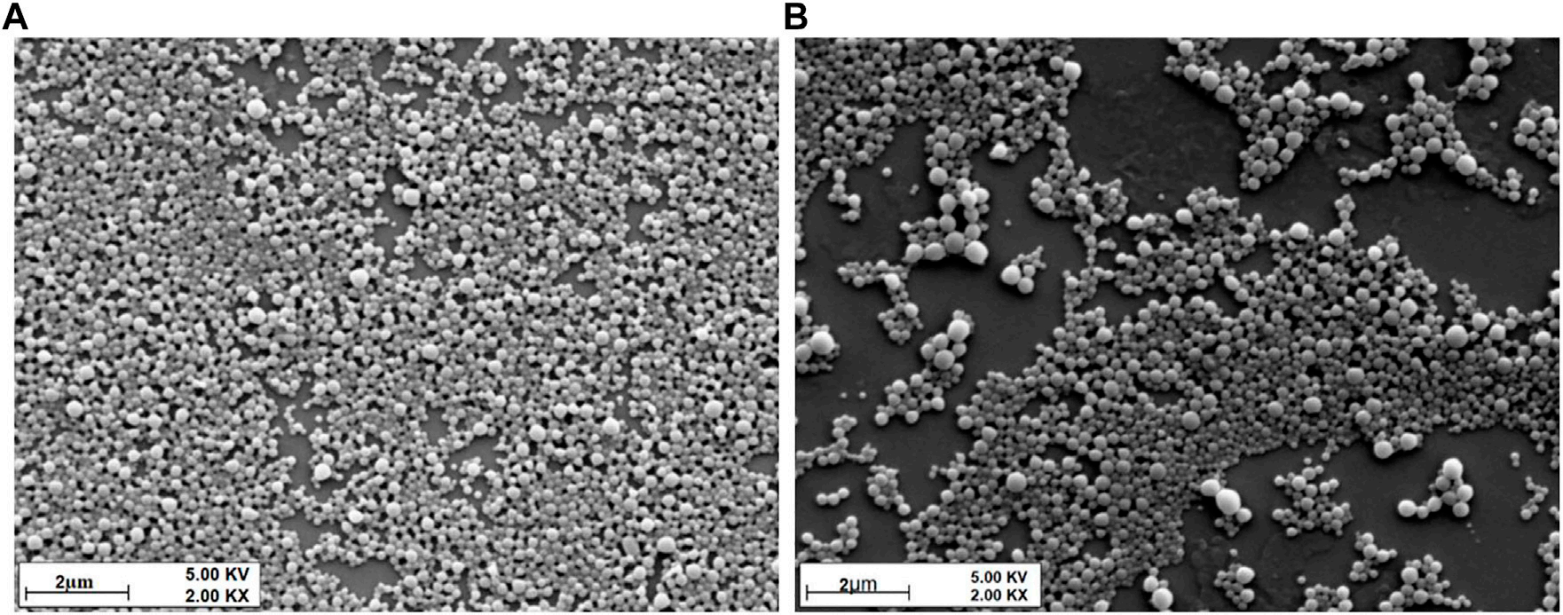
SEM micrographs of **(A)** 12.5% keto and 87.5% PLGA NP formulation and **(B)** 100% keto and 0% PLGA formulation.

The physicochemical properties of ketoconazole-loaded PLGA nanoparticles are reported in our previous article (Sadozai et al., 2020); briefly, a drug-to-polymer ratio was optimized in terms of particle size, PDI, and entrapment efficiency. It was observed that as the concentration of the drug increases, the particle size and PDI values increase. The maximum size was observed for formulation in which nanoparticles were prepared without PLGA polymer. The nanoparticles formulation containing 12.5% ketoconazole and 87.5% PLGA was chosen to prepare nanoparticle-laden gels since this formulation provides a sustained release of ketoconazole, shown in Figure 1. These nanoparticles are in the range of 150–200 nm, having discrete and homogeneous boundaries and no sign of agglomerations, as evident from SEM images (Figure 2), consistent with the data obtained from dynamic light scattering. However, the particles prepared with 100% drug are slightly larger.

#### 3.1.2 Scanning Electron Microscopy

The morphology, including the shape and surface of all the formulations, were studied by SEM. The images revealed uniformity and spherically shaped particles.

### 3.2 Optimization of Polymer Concentration for Preparation of Gel

Before the incorporation of nanoparticles, the gel formulation was optimized based on polymer concentration. Carbopol 934P-NF–grade polymer was used for the preparation of gels. The prepared gel formulations were examined visually for appearance and homogeneity. As shown in Table 5, all the formulations were clear and transparent with no particles. The clarity of gel plays an important role in acceptance and compliance. So, from an esthetic point of view, patients’ acceptability is important to achieve the desired results. The formulation containing 2% Carbopol 934P-NF showed excellent homogeneity without any lumps, as shown in Table 5, compared with other formulations. Formulation containing 2% Carbopol 934P NF showed acceptable physicochemical results at pH 6.5 compared with other formulations depicted in Table 5.

**TABLE 5:** Physicochemical characteristics of Carbopol 934P-NF gel base.

S. no.	Formulation	Appearance	*Homogeneity	Spreadability (mm)
1	1% Carbopol gel	Clear	***	Out of scale
2	1.5% Carbopol gel	Clear	***	Out of scale
3	2% Carbopol gel	Clear	***	28.33 ± 0.47
4	2.5% Carbopol gel	Clear	**	20.66 ± 0.47

*Homogeneity: * = Fair, ** = Good, *** = Excellent.

### 3.3 Incorporation of Nanoparticles in the Gel

Six different topical gel formulations were prepared, as shown in Table 6. Optimized ketoconazole-loaded PLGA nanoparticles (12.5% keto and 87.5% PLGA) were selected to incorporate w/w in the topical gel. This formulation was selected based on our previous studies, which showed extended release of drug for more than 24 h and the amorphous nature of the drug, which can enhance bioavailability at the site of infection. Similarly, silver nanoparticles were incorporated along with ketoconazole-loaded PLGA nanoparticles due to their synergistic effect. It was shown that silver nanoparticles could improve the efficacy of ketoconazole-loaded PLGA nanoparticles several folds when used together (Sadozai et al., 2020).

**TABLE 6:** Physicochemical characteristics of optimized gel formulations.

	Formulation	Appearance	*Homogeneity	Spreadability (mm)	**Drug content in gel (%)
1	Blank gel	Clear, opaque	***	28.33 ± 0.47	Nil
2	PLGA NP gel	Clear, opaque	***	26.01 ± 0.80	Nil
3	Keto NP gel	Clear, opaque	***	29.66 ± 1.24	2
4	Keto PLGA NP gel	Clear, opaque	***	26.33 ± 0.47	2
5	Keto-drug gel	Clear, opaque	***	33.33 ± 0.47	2
6	AgNP gel	Clear, half-white	***	34.66 ± 1.24	Nil
7	Keto PLGA NP + AgNP gel	Clear, half-white	***	32.00 ± 0.50	2
8	Commercial cream***	Clear, white	***	26.33 ± 0.47	2

*Homogeneity: * = Fair, ** = Good, *** = Excellent.

### 3.4 Physicochemical Properties of the Nanoparticle-Laden Gel

#### 3.4.1 Spreadability

The spreadability of gels prepared with different concentrations of Carbopol 934P-NF was also evaluated. At the lower composition of the polymer, the formulations failed the spreadability test due to low viscosity, whereas at higher composition, the spreadability of gel decreases. 2% Carbopol 934P NF was chosen as the optimum formulation for the preparation of nanoparticle-laden gels since it showed excellent spreadability properties, that is, 28.33 ± 0.47 mm. The spreadability of the gel was not markedly affected by the incorporation of nanoparticles (Table 6). The spreadability values were very close to the commercially available product that was found to have 26.33 ± 0.5 mm (Table 6).

### 3.5 *In Vitro* Drug Release Studies

**FIGURE 3:**
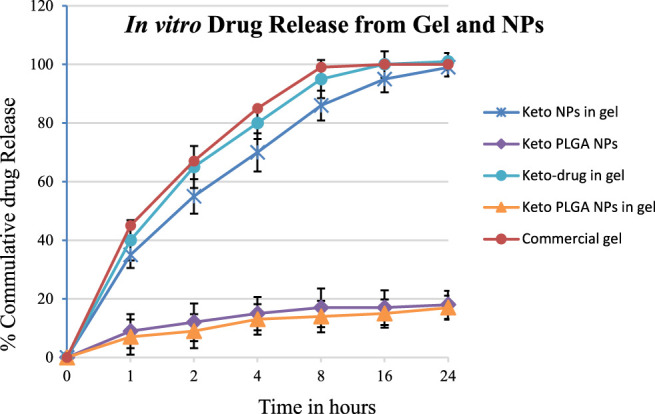
*In vitro* drug release of optimized gel formulations using Franz diffusion cell and optimized NPs formulation (12.5% keto + 87.5% PLGA NPs). Mean ± standard deviation (*n* = 3).

*In vitro* drug release studies were carried out using modified Franz diffusion cells together with cellulose membranes, as discussed in Section 2.6. The released drug amount obtained was plotted against time (Figure 3). The % cumulative drug released data which revealed that formulations keto-drug gel and keto NP gel release 35–40% of the drug in the first hour. In contrast, the optimized keto PLGA NP gel formulation releases 7% drug in the first hour. In comparison, the commercially available cream releases more than 45% of the drug. In 2 h, the optimized formulation releases 9% of the drug.

**FIGURE 4:**
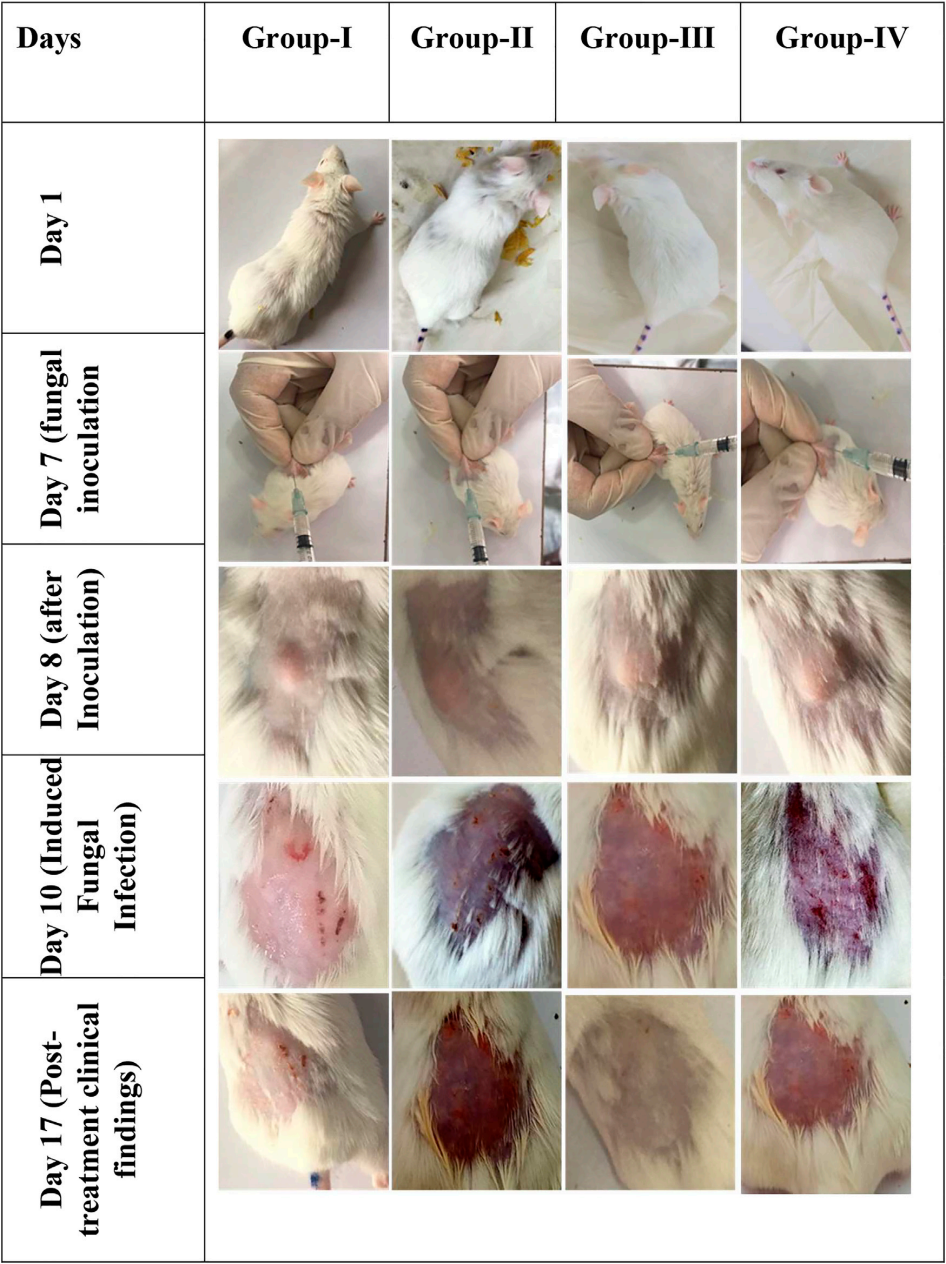
*In vivo* images of male Wister albino rats. Gel preparations were applied on the dorsal skin surface and observed for clinical findings (*n* = 6).

In contrast, keto-drug gel and keto NP gel released 55% and 65% of the drug, respectively, and commercially available cream releases up to 67% in the first 2 hours. The same pattern was observed, and 99% drug was released in the first 8 h from commercial cream, 95% from the keto-drug gel, 86% from keto NP gel, and 14% from keto PLGA NP gel. In the first 24 h, the optimized keto PLGA NP gel formulation released 17%, whereas all other formulations released more than 99% of the drug, as shown in Figure 4. These data suggested that not only Carbopol gel prolonged the drug release as compared to commercially available products, but the PLGA incorporating ketoconazole in the optimized formulation played an important role in delaying the drug release from the gel for several days (Chavan et al., 2021), which can be beneficial in reducing the frequency of gel application and availability of the drug for a longer period at the site of application. Similarly, keto PLGA NP gel provides an environment around the infectious skin where the drug is available for a longer period and extends the drug’s release (Waghule et al., 2020). The Carbopol gel acts as a reservoir in which the drug permeates and treats the targeted infectious area (Silva et al., 2015).

### 3.6 *Ex Vivo* Drug Penetration Studies

*Ex vivo* studies were carried out using human abdominal skin obtained from Caritas Klinik, Lebach, Germany. To determine the amount of drug that permeated from the gel through the skin, was retained on the skin, and deported in the skin layers, skin permeation studies, tape stripping method, and drug as discussed in detail in Section 2.7 were performed.

#### 3.6.1 Skin Permeation Studies

**FIGURE 5:**
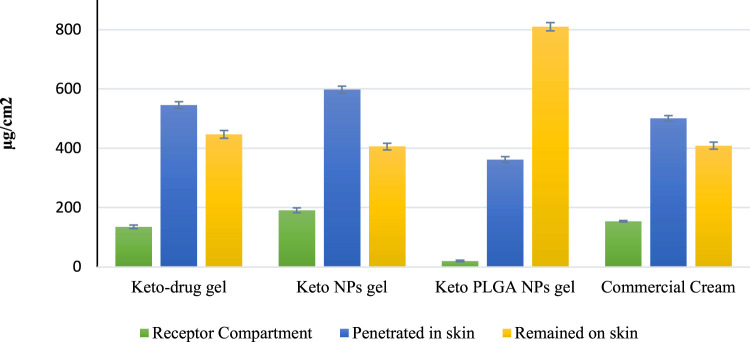
*Ex vivo* studies on the human abdominal skin. Comparison of drug concentration at different levels of human skin. Mean ± standard deviation (*n* = 3).

The data obtained from skin permeation studies, as shown in Figure 5, revealed that the maximum amount of drug permeated from commercially available cream through the skin compared with other gel preparations. Permeation profile indicated that 154 μg/cm^2^ (14.47%) of drug permeated through the skin from commercial ketoconazole cream, 135 μg/cm^2^ (11.96%) from keto-drug gel, and 191 μg/cm^2^ (15.98%) from keto NP gel, whereas it was 20 μg/cm^2^ (1.67%) from keto PLGA NP gel. The results obtained from skin permeation studies showed that our optimized keto PLGA NP gel formulation was successful in stopping the drug from permeation through the skin and only a negligible amount of drug permeated, which was less than 2% compared with the commercial cream from which more than 14% of drug permeated through the skin. Similarly, keto NP gel also showed higher permeation (15.98%) than Keto PLGA NP gel. The improved permeation of ketoconazole nanoparticles may be due to the particulate nature of ketoconazole. PLGA really retains the drug inside, thus not much material can permeate in case of ketoconazole-loaded PLGA nanoparticles. This might be due to the smaller size and larger surface area of the nanoparticles. It was also observed that PLGA polymer plays an important role in this nano-drug delivery system by providing a network-like structure in which the drug was uniformly distributed and difficult to permeate through the skin (Dinarvand et al., 2011). It was clear from these results that the optimized keto PLGA NP gel formulation is an ideal topical nano-drug delivery system that can hinder the permeation of the drug through the skin for a longer period and make it difficult to reach the systemic circulation. By controlling the skin permeation, our topical nano-drug delivery system could be used to minimize the systemic side effects co-related with these antifungal agents. Similarly, they can provide a better therapeutic reservoir on the skin to treat superficial infections effectively (Wu and Guy, 2009).

#### 3.6.2 Tape Stripping Method

The tape stripping method was performed to evaluate the amount of drug present on the surface of the stratum corneum using modified Scotch Magic Tape. The results obtained from HPLC data, as shown in Figure 5, demonstrated that the least amount of drug 406 μg/cm^2^ (33.97%) was present on the skin surface after applying the keto NP gel formulation. Similarly, keto-drug gel showed 447 μg/cm^2^ (39.62%) of the drug on the skin surface, and commercially available cream showed 409 μg/cm^2^ (38.42%) of the drug on the skin surface. Our optimized gel formulation showed excellent results as compared with the other gel formulations and commercial cream. Keto PLGA NP gel showed 810 μg/cm^2^ (67.97%) of the drug on the surface of the skin’s stratum corneum. For an ideal topical formulation to treat superficial infections, the drug should be on the skin’s surface for a longer period and in a larger amount. The results showed that optimized gel formulation could be used as a nano-drug delivery carrier because it showed less permeation and more drug retention on the skin’s surface.

#### 3.6.3 Drug Retention Studies

A drug retention study was performed to estimate the amount of drug retained inside the skin after 24 h. The results obtained from HPLC shown in Figure 5 demonstrated that for keto-drug gel, keto NP gel, and commercial cream, 48.40, 50.04, and 47.08% of the drug penetrated, respectively, which was far more than that from our optimized gel keto PLGA NP gel formulation (only 30.36% of the drug). The reason could be sustained release of drug from PLGA polymer within 24 h. Most of the drug remained on the surface (wrinkles and hair follicles) in the upper layer of the skin, unlike other gel preparations in which the drug release was not controlled and the drug permeated through various skin layers. These results also favored our optimized gel formulation for controlled and targeted drug release for a longer period. The PLGA played a dominant role in sustaining and retaining the drug on the skin and preventing permeation into the systemic circulation. We aimed to achieve maximum sustaining effect to avoid frequent application of the gel and minimize the drug dose. The problem of drug therapy failure could be easily addressed by applying our topical gel preparation.

### 3.7 *In Vivo* Evaluation of the Gel Against Fungal Infection

*In vivo* studies were carried out using male Wister albino rats approximately 230–250 mg in weight. All the animals were kept with care, and we strictly followed the ethical protocols. They were divided randomly into four groups, as shown in Table 7, and each group contained six male rats.

**TABLE 7:** Wister albino male rats’ group for *in vivo* studies.

**Group-I**	Positive control	Keto-drug gel
**Group-II**	Negative control	Blank gel
**Group-III**	Test drug	Keto PLGA NPs + AgNPs gel
**Group-IV**	Reference drug	Commercial cream

The infected area was treated with 1 mg of gel according to the group for 3 days, and we keenly observed the clinical parameters and effectiveness of applied gel on days 3, 5, and 7 as described in Table 4 and scored accordingly in Table 8.

**TABLE 8:** *In vivo* investigation of gel preparations and clinical findings.

S. no.	Group	Clinical finding	Score
1	Group-I	Slight erythematous skin, infection does not get cured completely	1
2	Group-II	Redness, loss of hair, and partial damage to the skin	4
3	Group-III	No sign of infection, hair regrowth started	0
4	Group-IV	Slight erythematous on the skin, infection does not get cured completely	1

It was observed that Group-II showed no sign of improvement, and clinical findings showed redness of the skin, loss of hair, and partial damage to the skin at the site of infection as it was treated with blank gel without any drug. Group-I and group-IV showed a little improvement compared to Group-II, and slight erythema was present, but the infection was not completely cured at the end of treatment of the infected area. Whereas Group-III showed maximum effectiveness from the gel preparation and no sign of infection was observed, our optimized keto PLGA NP + AgNP gel successfully cured the fungal infection as compared with the commercially available cream and keto-drug gel shown in Figure 4. The combination of ketoconazole-loaded PLGA NPs and AgNP-laden gel could be used as an ideal topical formulation to treat superficial skin infection caused by resistant strains of *C. albicans* and as a reference for those drugs having bioavailability and resistance issues.

## 4 Conclusion

This study supported the rational selection of polymer concentration to control the particle size and prolong the drug release for more than 24 h. Carbopol 934P-NF was used as a gel base; various formulations were prepared and their physicochemical properties were studied. Physicochemical characteristics of all the gel formulations were acceptable as compared with the commercial product. *In vitro* studies showed that keto PLGA NP gel formulation sustained the drug release for more than 24 h, whereas commercial cream and keto-drug gel released 99% drug in less than 24 h. *Ex vivo* studies supported our objective by limiting the permeation of drugs through the human abdominal skin compared to the commercial product and pure ketoconazole drug and maintaining a reasonable amount of drug at the skin’s surface to combat superficial fungal infection for a longer period. *In vivo* studies revealed that a combination of ketoconazole-loaded PLGA nanoparticles and AgNPs can effectively treat the fungal infection induced by a resistant strain of *C. albicans*. The combination of ketoconazole-loaded PLGA nanoparticles and AgNPs as a topical gel formulation could provide an opportunity to develop a cost-effective approach to achieve optimal therapeutic performance against resistant fungal infections at a much lower dose than is currently used. Additionally, it will enhance patient compliance by reducing the frequent application.

## Data Availability

The raw data supporting the conclusion of this article will be made available by the authors, without undue reservation.
